# Factors Affecting Green Agricultural Production Financing Behavior in Heilongjiang Family Farms: A Structural Equation Modeling Approach

**DOI:** 10.3389/fpsyg.2021.692140

**Published:** 2021-09-09

**Authors:** Hongli Wang, Shen Zhong, Jinguang Guo, Yu Fu

**Affiliations:** ^1^School of Public Administration, Dongbei University of Financel of Public Mana and Economics, Dalian, China; ^2^Institute of Finance, Harbin University of Commerce, Harbin, China; ^3^School of Public Administration, Dongbei University of Finance and Economics, Dalian, China

**Keywords:** family farm, green agricultural production, financing behavior, theory of planned behavior, SEM

## Abstract

Adhering to large-scale agricultural operations is one of the basic ways to develop green agriculture, and it is also an inevitable choice for the development of modern agriculture in the country. Among them, as a major agricultural production province in China, the development of family farms in Heilongjiang Province has a significant impact on green agriculture. Based on the theory of planned behavior (TPB), this study takes the 222-demonstration bases of family farms evaluated in Heilongjiang Province in 2019 as samples and constructs a structural equation model (SEM) to discuss the influence of participation in the family farms in green agricultural production financing behavior in-depth based on directional design, distribution, recycling, and sorting out questionnaires. The research found that the financing willingness of the farmers is determined by the attitude, subjective norms, and perceived behavior system of the family farm manager, and the financing willingness of the farmers and perceived behavioral control are determined by the financing behavior of the farmers. Among them, attitudes, subjective norms, and perceived behavioral control have a significant positive impact on financing intention and have a further effect on financing behavior. Financing willingness and perceived behavioral control have a direct effect on financial behavior and have a significant positive effect on it. This article aims to improve and enhance the financing environment for family farms to participate in green agriculture, to increase the enthusiasm of the new agricultural operators to participate in green agriculture.

## Introduction

China is a big agricultural country, and agriculture has always been in an important position in the economic development of China. Especially since the reform and opening-up, the agriculture of China has developed rapidly and experienced a series of major changes from collectivization to marketization, and small-scale peasant economy to a large-scale operation. However, due to many years of household contract responsibility system in China, coupled with insufficient social security, unclear land property rights, and other factors, the phenomenon of agricultural industry decentralization is serious (Ling et al., 2015). At the same time, it also brings a variety of environmental consequences that cannot be ignored (Chen et al., 2017; Shen et al., 2018; Liu Y. F. et al., 2020). Therefore, promoting agricultural modernization is of great significance to the sustainable development of agriculture of China, among which, insisting on large-scale agricultural management is one of the basic ways (Wan et al., 2018; Zhang et al., 2018).

The family farm as a new mode of large-scale operation, can effectively guarantee food security, increase the income of the farmers and narrow the gap between urban and rural, is the accelerator of agricultural economic development of China, and is also the development direction of future agricultural modernization. It not only promotes the process of agricultural commercialization, makes more scale and intensive agricultural production and operation but also contributes to improving the overall level of the agriculture (Mao et al., 2014; Vliet et al., 2015; Zhou et al., 2015; Graeub et al., 2016; Lowder et al., 2016; Veronika and Johannes, 2021). Green agriculture is based on “green environment,” “green technology,” and “green products,” which is transformed into a new agricultural development mode based on traditional agriculture. From the connotation of green agriculture, scholars mainly explain green agriculture from two perspectives. The first is that green agriculture pays full attention to the relationship between man and nature and pays more attention to the harmonious development of man and nature (Ghadiyali and Kayasth, 2012). The second is that green agriculture should follow the environmental law, make rational use of agricultural resources, and rely on green technology to realize the green transformation of agricultural economic activities (Behera, 2012). No matter from the perspective of factors of production such as land, capital, and labor, or from the point of view of product attributes and development concept, family farms are more tend to kind of green agricultural enterprises. Compared with traditional farmers, the economic strategy of the family farm is oriented to consumers, the market, and the future. It emphasizes more enterprise and scale management, pays more attention to the brand marketing concept and agricultural product certification, and, therefore, attaches more importance to the development of family farm green development (Gao et al., 2017a).

However, the family farms are still in their infancy, and there are many problems in the development process (Guo, 2013; Du and Xiao, 2014). On the one hand, in the early stage of development, the operators need to savings, scale, specialization of management to lay a solid foundation, solve the unexpected needs of land circulation and scale expansion. On the other hand, rural financial institutions, as indispensable capital suppliers of new agricultural operation entities, have not been able to adapt to the new operation mode of family farms in terms of credit products and financial services, and the serious information asymmetry has caused the mismatch between credit demand and supply (Xu, 2014; Gao et al., 2017b). Therefore, the financing problem is the main dilemma faced by family farms in the process of green development, which hinders the growth of family farms to a large extent and seriously restricts the pace of agricultural modernization (Howley et al., 2014). Financial support can promote the further development of family farms by affecting farmers’ psychological structure (attitude, subjective norms, perceived behavioral control) to determinine their financing behavior (King and Levine, 1993; Morck and Nakamura, 1999; Cafer and Rikoon, 2018; Stefan et al., 2018). Knis et al. (2016) divided farmers into poor type, maintenance type, and affluent type in the study, and found that among the three types of farmers, the affluent type had the strongest financing willingness, followed by the maintenance type, while the poor farmers had a very negative attitude toward borrowing from financial institutions, and their financing willingness was very weak. They said that even if they could borrow by the mortgage, they are also reluctant to mortgage with financial institutions, because they often default due to lack of integrity, and the collateral cannot be recovered, so for them, the loss of high-value collateral is greater. In addition, this study proposes that education level, income level, vocational training, and other factors can also affect the attitude of the farmers and willingness to determine their financing behavior. Hansson et al. (2012) believed that attitude is the most important psychological activity in decision-making, and farmers make cognitive choices based on attitude and subjective norms. Kuhfuss et al. (2016) also reached a similar conclusion and emphasized the influence of social norms, that is, individual behavior is easily affected by other individual behaviors. Therefore, it is very important to analyze the psychological factors that affect the financing behavior of family farms for the green development of agriculture.

Heilongjiang Province, as a major agricultural production province in China, has become the most important commodity grain production base in China, and its agricultural operation has gradually become large-scale. However, due to the relatively backward level of financial development, the financing problem of family farms in the Heilongjiang Province is more prominent. Therefore, it is of great significance to study the influencing factors of family farms participating in green agricultural production financing behavior of Heilongjiang Province to promote the sustainable development of agriculture in China.

The existing research has achieved a wealth of research results, which has laid a solid foundation for this study to research family farm financing behavior but there are still some shortcomings: First, the existing literature on agriculture mainly focuses on traditional agriculture with economic benefits, and the degree of attention to environmental protection is insufficient. Second, the research on the financing behavior of the farmers only stays at the national level, lacking the research on the financing behavior of the farmers in provinces, especially in some representative provinces. Third, the research subjects mainly focus on ordinary small-scale farmers, ignoring the important impact of the financing behavior of family farms, a new agricultural management subject, on the development of green agriculture. In addition, the existing literature in the study of financing behavior of the farmers is based on subjective consciousness to select variables and lack of necessary theoretical basis. Therefore, the contributions of this study are as follows: first, there are many family farms in China, among which the Heilongjiang Province is a big agricultural province, and its family farm demonstration field has a typical representative role. This study takes 222 demonstration bases of family farms in Heilongjiang Province as the research object for the first time to analyze their financing behavior. Second, based on the research perspective of the demonstration base of the family farm, this study constructs the research framework of the family farm and green agricultural production. Third, based on the theory of planned behavior (TPB), this study uses TPB to test the potential psychological structure and studies the financing behavior of family farms participating in green agricultural production, and to analyze the potential factors influencing the diversification of financing behavior of family farms. At the same time, the structural equation model (SEM) is used to explore the influencing factors of participation of the family farms in green agricultural production financing behavior and put forward the countermeasures and suggestions to optimize the financing environment of the demonstration base of family farms participating in green agricultural production.

## Theoretical Framework and Research Hypothesis

### Theoretical Framework

**FIGURE 1:**
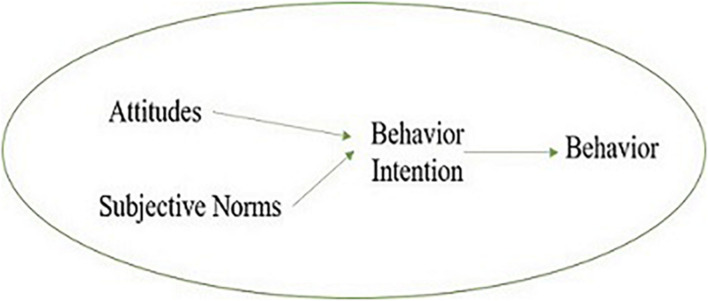
Conceptual model of rational behavior theory.

Ajzen and Fishbein (1980) pointed out that rational behavior theory is a theoretical model to understand and predict human behavior. According to this theory, human is rational, and individual behavior is mainly determined by individual behavioral intention, that is, the intensity of willingness of the individual to carry out a certain behavior. Therefore, to some extent, individual behavior can be predicted by individual behavior intention, which in turn depends on individual attitudes and subjective norms. Among them, the attitude of the individual to behavior refers to the perception of the individual and evaluation of the possible results of a certain behavior; the subjective norm refers to the perception of the individual of the opinions of the important people or groups on their behavior, and the motivation to keep consistent with the opinions of these people or groups. The conceptual model of rational behavior theory is shown in Figure 1.

**FIGURE 2:**
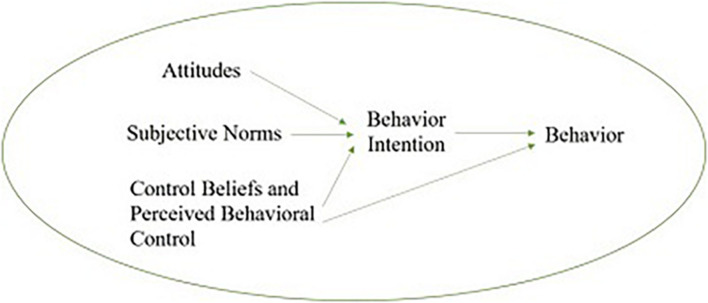
Conceptual model of planned behavior theory.

The theory of rational behavior holds that behavior is only controlled by will. However, many behaviors are not only controlled by will but also influenced by other factors. Taking family farm financing as an example, whether the farmer carries out financing is largely affected by the degree of financing willingness, but it is also restricted by subjective and objective factors such as self-owned capital, financing interest rate, education level, and financing channels. Therefore, the theory of rational behavior is not suitable to predict the behavior which is controlled by unwillingness. Therefore, the TPB is based on the theory of rational behavior, adding the element of control beliefs and perceived behavioral control (Ajzen, 1985), and using it as a substitute variable of actual constraints to predict the possibility of behavior. In addition, TPB further points out that if a behavioral intention of the person is stronger, he is more likely to make a certain behavior, that is to say, the behavioral intention is the direct precursor of a specific behavior (Ajzen and Fishbein, 2005). The specific conceptual model diagram is shown in Figure 2.

In view of this, this study analyzes the influencing factors of the demonstration base of family farms participating in green agricultural production financing behavior from four aspects of behavior attitude, subjective norms, perceived behavioral control, and behavior intention.

### Research Hypothesis

According to the TPB, the attitude of the individual refers to the perception and evaluation of the possible results of certain behavior. TPB can not only effectively predict behavior but also provide an effective framework for behavior change (Chase, 2014). Beedell and Rehman (2000) pointed out that attitude plays a decisive role in the decision-making of the farmers. Therefore, in the process of financing decision-making, the attitude of the farmers has a significant impact on their final financing willingness. Different financing motives reflect the different views of family farmers on financing behavior. We can deduce their attitude toward financing behavior from different financing motives of family farmers. Combined with the research of Ploypailin (2021), this study evaluates the attitude of the family farm to participate in the financing behavior of green agricultural production from three aspects, namely, the expectation of family farm operators on the economic benefits of the farm operation, the expectation of green agricultural development, and the psychological expectation of the incentive policies of the government. Based on this, the hypothesis is put forward:

H1: Attitude of the farmers have a significant positive impact on their willingness to participate in green agriculture financing.

Subjective norms mainly reflect the social pressure on individuals when they take certain actions. Xu et al. (2014) pointed out that if other people expect them to make a decision, the individual will also think that the action is feasible. Chen (2016) and Nadia et al. (2018) reached similar conclusions. In terms of subjective norms, the decision-making of the farmers is mainly affected by the opinions of family members, relatives and friends, and regional entrepreneurial climate (Yanto et al., 2016; Al Balushi et al., 2018). Therefore, this study measures the subjective norms of family farms participating in green agricultural production financing behavior from three aspects, namely, whether family members support it, whether relatives and friends support it, and the financing atmosphere in the region. Based on this, the hypothesis is put forward:

H2: Subjective norms of the farmers have a significant positive impact on their willingness to participate in green agriculture financing.

Perceptual behavioral control can not only affect the behavior intention of the individual indirectly but also directly (Ajzen, 1991, 2005). Taylor and Todd (1995) believed that there is a positive relationship between perceived behavior control and behavior intention. Farrell et al. (2016) reached a similar conclusion and pointed out that perceived behavior control is one of the important factors affecting decision-making. Therefore, a comprehensive analysis of the losses and benefits obtained by financing behavior of the farmers is not only conducive to the correct judgment of financing behavior of the farmers choice but also conducive to the in-depth discussion of the researchers on the motivation mechanism of financing behavior. According to the cost-benefit theory, the cost of participation of the farmers in green agriculture financing consists of direct cost and indirect cost. The direct cost mainly includes three aspects, namely, capital cost, time cost, and credit cost; the indirect cost mainly includes the risk cost caused by financing (Freeman et al., 2014; Bergstrom and Randall, 2016). In this study, the influencing factors of perceived behavioral control on the participation of the farmers in green agriculture financing can be explained from the following four aspects, namely, the interest level of loans, the efficiency of financing, whether there is collateral, and repayment risk. In addition, the TPB theory further points out that behavior intention refers to the subjective probability of a decision-maker to make a certain behavior. The higher the probability, the greater the possibility of an individual to implement the behavior. In other words, the willingness of the farmers to finance is an important incentive for farmers to generate financing behavior. The stronger their willingness, the more likely their financing behavior is to occur (Al Balushi et al., 2018; Ploypailin, 2021). Based on this, the hypothesis is put forward:

H3: Perceived behavioral control of the farmers has a significant positive impact on their willingness to participate in green agriculture financing.

H4: Perceived behavioral control of the farmers has a significant positive impact on their participation in green agriculture financing.

H5: The financing willingness of the farmers has a significant positive impact on their participation in green agriculture financing behavior.

## Materials and Methods

### Design and Sample

Brown and Cantor (2000) proposed that a structured questionnaire can collect a relatively large amount of data in a short time, so this study uses a questionnaire survey to obtain research data. In 2019, in order to give full play to the demonstration and guidance role of farmers’ family farms, Heilongjiang province adopted the procedures of county-level application for preliminary review, municipal review and verification, and expert review and identification. After being reviewed and approved by the office meeting of the director of the Provincial Agricultural Commission, 222 provincial farmers’ family farm demonstration farms engaged in green agricultural production were selected in the province. In this study, 222 family farms were taken as research samples, and with the help of the Heilongjiang Provincial Department of agriculture, questionnaires were sent to them. The questionnaire (Appendix 1) consists of two parts. According to the research of Li et al. (2020), the previous part of the survey includes seven aspects, namely, gender, age, education level, operation time of the farm, annual income of the farm, annual expenditure of the farm, and whether it has been financed before; the second part, adapted from Borges et al. (2014) and Nadia et al. (2018), analyzes the influencing factors of participation of the family farms in green agriculture financing behavior from five aspects, namely, behavior attitude, subjective norms, perceived behavioral control, financing willingness, and financing behavior. Likert’s five scales were used in the questionnaire, with scores of 1, 2, 3, 4, and 5 representing complete disagreement, disagreement, general agreement, basic agreement, and complete agreement (Harpe, 2015). The final formal questionnaire includes five potential variables and 18 measurement items (Table 2). In addition, the effective rate of the questionnaire is 100%, the samples meet the requirements of the SEM model, the research data is relatively reliable, and has a certain policy conversion value (Chen et al., 2012; Molwus et al., 2017).

**TABLE 1:** Basic information of farmers interviewed.

Index	Category	Frequency	Percentage	Effective percentage	Cumulative percentage
Gender	Male	139	62.6	62.6	62.6
	Female	83	37.4	37.4	100.0
Age of farmers	<30	43	19.4	19.4	19.4
	31–45	47	21.3	21.3	40.8
	46–60	55	24.6	24.6	65.4
	>60	77	34.6	34.6	100.0
Education level of farmers	Junior high school and below	61	27.5	27.5	27.5
	High school	84	37.9	37.9	65.4
	Junior college	28	12.4	12.4	77.8
	Undergraduate	22	10.0	10.0	87.8
	Master degree or above	27	12.2	12.2	100.0
	5–10 year	55	24.8	24.8	86.9
	>10 year	29	13.1	13.1	100.0
	210–300 thousand	14	6.3	6.3	33.3
	310–400 thousand	56	25.2	25.2	58.5
	41–50 thousand	75	33.8	33.8	92.3
	>500 thousand	17	7.7	7.7	100.0
Annual farm expenditure	<50 thousand	33	14.9	14.9	14.9
	60–100 thousand	41	18.5	18.5	33.4
	110–150 thousand	77	34.7	34.7	68.1
	160–200 thousand	46	20.7	20.7	88.8
	>200 thousand	25	11.2	11.2	100.0
Has the farm ever been financed	Yes	77	34.6	34.6	34.6
	No	145	65.4	65.4	100.0

**TABLE 2:** Reliability test results.

Dimension	Subject	Global Cronbach’s α	Global Cronbach’s alpha
Attitude	Q1	0.803	0.846
	Q2		
	Q3		
Subjective norm	Q4	0.821	
	Q5		
	Q6		
Perceived behavior control	Q7	0.863	
	Q8		
	Q9		
	Q10		
Financing willingness	Q11	0.739	
	Q12		
	Q13		
Financing behavior	Q14	0.725	
	Q15		

### Data Description and Analysis

Among the 222 demonstration farms investigated in this study (Table 1), 62.6% of the total sample gender of the farmers are men and 37.4% are women. It can be seen that most families are still headed by men, and there are traces of a small-scale peasant economy in rural agricultural production. The proportion of farmers over 60 years old was the highest, accounting for 34.6%, and the proportion of farmers aged 46–60 years old was the second, accounting for 24.6%, accounting for more than 50%. The proportion of children aged 31–45 and under 30 years old are 21.3 and 19.4%, respectively. It can be seen that the older you are, the more experienced you are in family farm production and the better the farm management. In the survey, 37.9% of the family farmers have a high school education, 27.5% have a junior high school education, and 12.4% have a junior college education. Nearly 80% of the farmers have a degree of bachelor or below. It can be seen that the family farmers generally have low education. In the survey of demonstration farms, the proportion of farms with 3–5 years of operation is the highest, accounting for 32.4%, followed by 1–3 years and 5–10 years, accounting for 29.7 and 24.8%, respectively, and the proportion of farms with more than 10 years is the lowest, accounting for 13.1%. It can be seen from the data that the operation of the family farm is a bottleneck period in about 5 years. At this time, the farm needs more financial support to expand production. If there is a continuous lack of funds, the production situation of the farm will decline year by year. According to the statistics of farm income, the middle-aged income of the demonstration farm is the most in the range of RMB 410–500 thousand, accounting for 33.8%. The income below RMB 20 thousand and 31–40 thousand rank second and third, accounting for 27.0 and 25.2%, respectively. The income above RMB 50 thousand and 21–30 thousand is the least, accounting for 7.7 and 6.3%. Among the 222 demonstration farms, more than 70% of them have an annual income of more than RMB 20 thousand. It can be seen that the income of family farms engaged in green agricultural production is still considerable. According to the survey of farm annual income, we find that 34.7% of the farms with the annual expenditure of RMB 11–15 thousand accounted for the highest proportion, while 20.7% of the farms with an annual expenditure of RMB 16–20 thousand and 11.2% of the farms with the annual expenditure of more than RMB 20 thousand accounted for the highest proportion. Nearly 70% of the annual expenditure of the farm is more than RMB 10 thousand, which indicates that the demonstration farm has a large demand for funds in the process of green agricultural production, which is also in line with the original intention of this study. Finally, in the survey on whether the farms have been financed, 65.4% of the farms have not been financed, and only 34.6% of the farms have been financed. It can be seen that there are still more farmers who have not made any financing due to various factors. On the other hand, we can also see that there is still a huge space for the development of family farms in Heilongjiang Province, and the shortage of funds is the primary problem we need to solve.

## Results

### Reliability Analysis of the Scale

Reliability refers to the reliability of measurement results, and its significance refers to the consistency and stability of measurement values. The main methods to measure the reliability are test-retest reliability, half reliability, and internal consistency reliability. Internal consistency reliability is a commonly used method to evaluate the reliability of the scale. Cronbach’s α is used to indicate the degree of reliability. Generally speaking, when the α coefficient is greater than 0.7, the reliability of the questionnaire is better (Liu J. et al., 2020). In this study, SPSS26.0 software is used to test the reliability of the questionnaire data. As shown in Table 2, the overall Cronbach’s α coefficient of 222 valid questionnaires is 0.846. The overall Cronbach’s α coefficients of behavioral attitude, subjective norm, perceived behavior, financing willingness, and financing behavior were 0.803, 0.821, 0.863, 0.739, and 0.725, respectively. Therefore, the reliability of this questionnaire is good and the reliability is high.

### Validity Analysis of the Scale

Validity analysis refers to the degree of a certain attribute that can be measured by a questionnaire or scale. The more the result of the questionnaire is consistent with the real situation of a certain attribute, the higher the validity of the questionnaire; on the contrary, the lower the validity of the questionnaire.

#### Exploratory Factor Analysis

**FIGURE 3:**
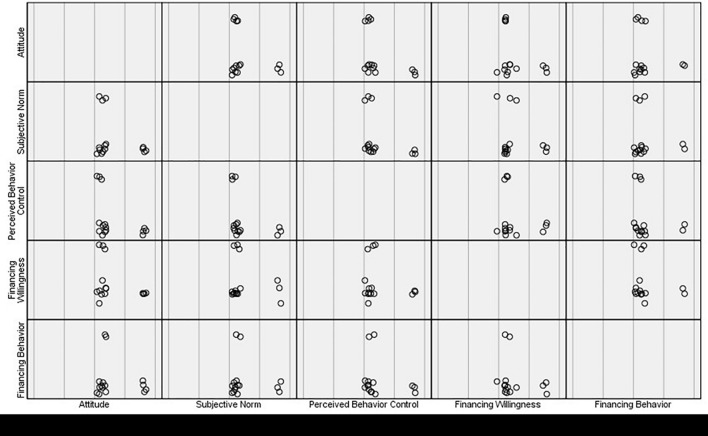
Matrix diagram.

To ensure that the data of the questionnaire can accurately measure the behavior attitude, subjective norms, and perceived behavioral control of family farm operators, this study uses SPSS26.0 to further analyze the validity of the questionnaire and uses the exploratory factor analysis method of structural validity to test the validity. The purpose of exploratory factor analysis is to reduce many observed variables to a few factors. Before exploratory factor analysis, it is necessary to test whether the survey data are suitable for factor analysis. Among them, the commonly used test indexes are kaiser-meyer-olkin (KMO) value and Bartlett’s test of sphericity. The closer the KMO value is to 1, the stronger the correlation between variables. It can be seen from Table 3 that the KMO value of the data is 0.809 (>0.8), *p* = 0 (<0.05), indicating that the observation indexes are suitable for factor analysis. Therefore, this study makes exploratory factor analysis on all topics. First, principal component analysis is used to extract common factors, and the factors with eigenvalues greater than 1 are extracted during analysis. The maximum variance method is used to rotate the factors to verify the classification of indicators and define the factors. The analysis results and gravel diagram are shown in Table 4 and Figure 3. The results show that after the principal component analysis, five factors with eigenvalues greater than 1 are extracted according to the principle of eigenvalues greater than 1, which is consistent with the dimension design of the scale. The cumulative variance contribution rate is 72.39%, which can cover most of the information on the scale. It also shows that the factor extraction result is ideal.

**TABLE 3:** KMO and Bartlett test results.

KMO		0.809
Bartlett sphericity test	Approximate χ^2^	1317.460
	Degree of freedom	105
	Significance	0.000

**TABLE 4:** Results of principal component analysis.

Component	Initial eigenvalue			Extract the load sum of squares			Sum of squares of rotational loads		
	Total	Variance percentage	Accumulate %	Total	Variance percentage	Accumulate %	Total	Variance percentage	Accumulate %
1	4.9	32.669	32.669	4.9	32.669	32.669	2.871	19.139	19.139
2	1.952	13.01	45.679	1.952	13.01	45.679	2.199	14.657	33.796
3	1.679	11.195	56.874	1.679	11.195	56.874	2.189	14.595	48.391
4	1.245	8.298	65.172	1.245	8.298	65.172	2.037	13.577	61.968
5	1.082	7.216	72.388	1.082	7.216	72.388	1.563	10.42	72.388

It can be common factors of attitude, subjective norm, perceived behavioral control, financing willingness, and financing behavior have good explanatory power.

#### Confirmatory Factor Analysis

**FIGURE 4:**
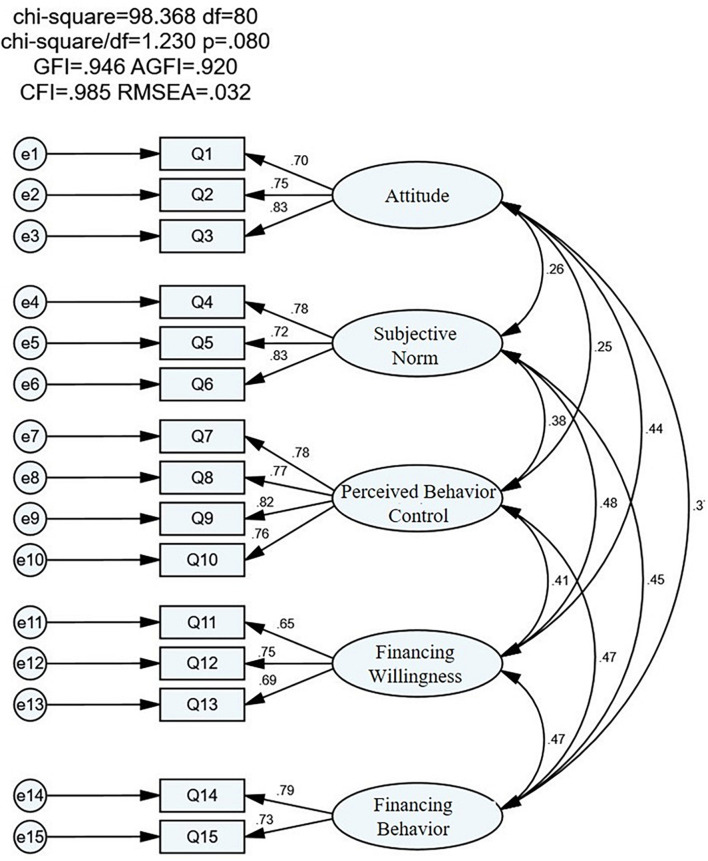
Confirmatory factor analysis (CFA) model diagram.

The purpose of Confirmatory Factor Analysis (CFA) is to test whether the observed indicators can effectively measure their corresponding factors through survey data, that is, to test the fitting ability of the preset factor model (Anderson and Gerbing, 1988). Therefore, CFA is used to test the construct validity, convergent validity, and discriminant validity of the scale. The results are shown in Figure 4 and Tables 5–7. As shown in Figure 4 and Table 8, χ^2^/degree of freedom (DF) = 1.23, root mean square error of approximation (RMSEA) = 0.032, goodness-of-fit index (GFI) = 0.946, adjusted goodness-of-fit index (AGFI) = 0.92, comparative fit index (CFI) = 0.985, incremental fit index (IFI) = 0.986, tucker-lewis index (TLI) = 0.981, it shows that the CFA model has a good fit, and all indicators are within the standard range.

**TABLE 5:** Results of discriminant validity analysis.

	Perceived behavior control	Subjective norm	Attitude	Financing willingness	Financing behavior
Perceived behavior control	0.763				
Subjective norm	0.150	0.779			
Attitude	0.120	0.215***	0.783		
Financing willingness	0.151***	0.195***	0.138***	0.700	
Financing behavior	0.171***	0.250***	0.217***	0.159***	0.760

*** denote statistical significance at the 1% significance levels.

**TABLE 6:** Fitting results of structural equation model.

Classification	Absolute fitting index				Value added fitting index				Parsimony fit index	
Index	CMIN/DF	GFI	AGFI	RMSEA	NFI	RFI	IFI	CFI	PCFI	PNFI
Fitting value	1.298	0.942	0.915	0.037	0.921	0.899	0.981	0.980	0.766	0.720
										

**TABLE 7:** Path analysis.

Path			Standardization coefficient	C.R.	*p*
Financing willingness	←	Attitude	0.228	3.648	***
Financing willingness	←	Subjective norm	0.206	3.762	***
Financing willingness	←	Perceived behavior control	0.140	2.287	0.022
Financing willingness	←	Financing willingness	0.522	3.860	***
Financing behavior	←	Perceived behavior control	0.313	3.558	***

*** denote statistical significance at the 1% significance levels.

**TABLE 8:** Results of confirmatory factor analysis.

Index	X2/df	RMSEA	GFI	AGFI	CFI	IFI	TLI
	1.23	0.032	0.946	0.92	0.985	0.986	0.981

As shown in Table 9, the standardized factor load coefficient values of each observation index of convergent validity on its corresponding latent variables are greater than 0.5, the combined reliability CR values of each variable are greater than 0.7, and the average variance extraction (AVE) values of each variable are greater than 0.5, which meet the standard requirements of convergent validity. Therefore, the scale has good convergent validity.

**TABLE 9:** Standardized regression coefficients and standard errors for latent variable pathways.

Path			Estimate	AVE	CR
Q1	←	Attitude	0.701	0.5819	0.806
Q2	←	Attitude	0.752		
Q3	←	Attitude	0.83		
Q4	←	Subjective norm	0.781	0.6071	0.8221
Q5	←	Subjective norm	0.724		
Q6	←	Subjective norm	0.829		
Q7	←	Perceived behavior control	0.777	0.6136	0.8639
Q8	←	Perceived behavior control	0.773		
Q9	←	Perceived behavior control	0.82		
Q10	←	Perceived behavior control	0.762		
Q11	←	Financing willingness	0.653	0.4897	0.7416
Q12	←	Financing willingness	0.749		
Q13	←	Financing willingness	0.694		
Q14	←	Financing behavior	0.788	0.5769	0.7314
Q15	←	Financing behavior	0.73		

It can be seen from Table 5 that the AVE square root of each latent variable in the discriminant validity test is greater than the correlation coefficient with other latent variables, which indicates that there is good discrimination between the observation indexes measuring different latent variables, and the discriminant validity of the scale in this study has also been effectively guaranteed.

To sum up, it shows that the sample quality of this survey is good, the data is more effective and the answer of the respondents is reliable.

### Test of the Structural Model

**FIGURE 5:**
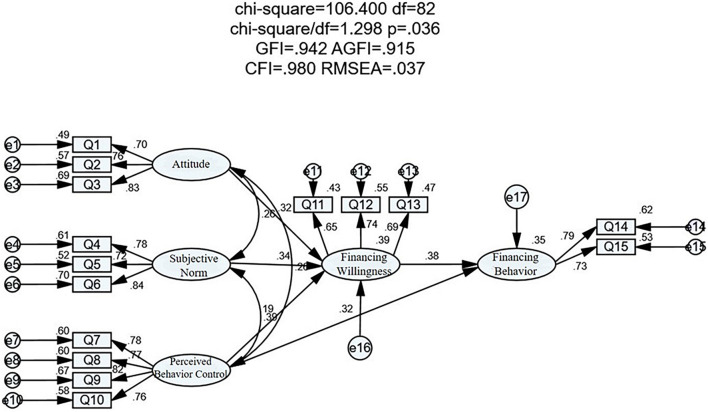
Execution results in structural equation model (SEM).

In this study, AMOS22.0 is used to estimate and test the (SEM) established in this study by using the maximum likelihood method, to ensure that the fitting index of each SEM meets the fitting evaluation standard. The model implementation results are shown in Figure 5.

According to Table 6, the absolute fitting index CMIN/DF = 1.298 (<3), the relative fitting index GFI = 0.942 (>0.9), AGFI = 0.915 (>0.9), RMSEA = 0.037 (<0.05), and all the indexes fit well; in the value-added fitting index, NFI = 0.921, RFI = 0.900, IFI = 0.981, CFI = 0.980 are all greater than the critical value of 0.9, which fully meets the fitting standard; in the simple fitting index, NFI = 0.921, RFI = 0.900, IFI = 0.981, CFI = 0.980 are all greater than the critical value of 0.9, PGFI = 0.644 (>0.5), PNFI = 0.720 (>0.5), all meet the standard.

### Path Analysis

This study uses path analysis to further verify the above hypothesis (Table 7). As can be seen from the results, the standardized path coefficient is 0.228 (*p* < 0.001), which is significant at the significant level of 1%, indicating that the attitude of family farmers has a significant positive impact on their financing willingness. The path coefficient of subjective norms affecting financing willingness is 0.206 (*p* < 0.001), which is significant at a 1% significance level, showing that the subjective norms of family farmers have a significant positive impact on their financing willingness. The path coefficient of perceived behavior influencing financing willingness is 0.140, *p* = 0.022 (<0.05), which indicates that the perceived behavior of family farmers has a significant positive impact on their financing willingness. The path coefficient of financing willingness influencing financing behavior is 0.522 (*p* < 0.001), indicating that the financing willingness of family farmers has a significant positive impact on their financing behavior. The path coefficient of perceived behavior influencing financing behavior is 0.313, and *p* < 0.001, which shows that the perceived behavior of family farmers has a significant positive impact on their financing behavior.

### Mediating Effect Test

In addition, considering that there may be mediating effect in the influencing factors of participation of the family farmers in green agricultural production financing behavior, this study uses AMOS22.0 software to conduct a further study by bootstrap test. Among them, the random sample set is 2,000, and the confidence interval (CI) is 95%. According to Table 10, the CI of behavior attitude → financing willingness → financing behavior is [0.033, 0.250], and the mediating effect coefficient is 0.119. The CI of subjective norm → financing willingness → financing behavior is [0.027, 0.247], and the mediating effect coefficient was 0.107. The CI of perceived behavioral control → financing willingness → financing behavior is [0.001, 0.221], and the mediating effect coefficient was 0.073. The CIs of the three paths are both positive numbers, excluding 0. Therefore, the mediating effect of financing willingness on the participation of the household farmers in green agricultural production financing behavior is significant.

**TABLE 10:** Mediating effect test results.

Path	Standardized path coefficient	Bootstrap		*p*
		Lower	Upper	
Attitude → Financing willingness → Financing behavior	0.119	0.033	0.250	0.003
Subjective norm → Financing willingness → Financing behavior	0.107	0.027	0.247	0.005
Perceived behavior control → Financing willingness → Financing behavior	0.073	0.001	0.221	0.005

## Discussion

The attitude of family farm operators has a significant positive impact on their willingness to participate in green agricultural production financing, thus affecting the financing behavior of the farmers (Läpple and Kelley, 2013; Veronika et al., 2020) used the discrete choice experiment to investigate the willingness of German farmers to accept sustainability standards, and found that the behavior and attitude of the farmers have a significant impact on the final decision-making. In the TPB, the process of attitude formation is the process of attitude acquisition. The relationship between attitude and behavior is extremely close, and there is a high degree of consistency between individual attitude and behavior. Therefore, if the family farmers think that financing can obtain more income, financing is more conducive to the development of green agriculture, and government policies are more conducive to their financing, then the willingness of the farmers to finance is stronger, which is easier to produce financing behavior.

The subjective norms of family farm operators also have a significant positive impact on their willingness to participate in green agricultural production financing, thus affecting the financing behavior of the farmers. The observation of Hansson et al. (2012) also supports this result, that is, the psychological structure of the people can affect the decision-making of the farmers. Human beings are social animals with social attributes. Living in a society, people cannot escape from social life and the influence of the surrounding environment on their behavior, that is, there will be a certain “neighbor effect” (Chabé-Ferret et al., 2018; Le Coent et al., 2018). The family members, relatives, and friends of farmers, and the cultural atmosphere of their living environment are all important variables that affect the financing willingness of the farmers. In other words, subjective norms of the farmers change their financing willingness through the process of internalization and identification, thus affecting their financing behavior. Therefore, the more support a farmer gets from his family and friends, the stronger the financing atmosphere in his area, the stronger the willingness of the farmer to finance, and the easier it is to generate financing behavior (Nadia et al., 2018).

Perceived behavioral control of the farmers has a significant impact on financing willingness and financing behavior. On the one hand, perceived behavioral control factors indirectly affect financing behavior by influencing financing willingness. On the other hand, perceived behavioral control can directly affect financing behavior. In this study, perceived behavioral control refers to the subjective judgment of family farm operators on whether they can carry out financing behavior. It reflects the perception of the farmers of individual internal factors and environmental external factors of the specific goal of financing behavior. The stronger the perceived behavioral control of the farmers, the stronger the financing willingness, and the easier it is to carry out financing behavior. Therefore, we can further find that the lower the interest level of the loan, the higher the financing efficiency, the more sufficient the collateral, and the smaller the repayment risk, the stronger perceived behavioral control. On the one hand, it directly affects their financing behavior, on the other hand, it indirectly affects their financing behavior by affecting their financing willingness. The interaction of the two is more likely to lead to financing behavior (Liu et al., 2018).

## Conclusion and Implication

In this study, 222 provincial household farm demonstration farms selected by Heilongjiang Province in 2019 are selected as research samples, and questionnaires are designed, distributed, and collected. Based on the SEM, the influencing factors of household farm demonstration farms participating in green agricultural production financing behavior in Heilongjiang Province are investigated. The results show that the attitude and subjective norms of family farm operators have a significant positive impact on their willingness to participate in green agricultural production financing, and further indirectly affect the financing behavior of the farmers. Perceived behavioral control of the farmers has a significant impact on financing willingness and financing behavior. On the one hand, perceived behavioral control factors indirectly affect financing behavior by influencing financing willingness; on the other hand, perceived behavioral control can directly affect financing behavior.

Based on the above conclusions, we propose the following suggestions:

In terms of national policies, we should establish and improve the agricultural policy financial system, improve the farmland financial system, and promote the financial needs of farmers. In addition, the government should increase the support for informal financial channels, give preferential policies, speed up the development of various forms of rural financial organizations, and guide and regulate informal financing, so that the formal financing channels and informal financing channels can coexist and accommodate each other, and improve the new rural financial system.

For financial institutions, it is necessary to optimize the credit business process, simplify the loan operation procedures, and improve the efficiency of loan processing of the farmers. On the one hand, it is to simplify the process of handling business, strengthen the standardized operation of a business, and reduce the time cost of financing. On the other hand, in the process of customer business investigation, we should deepen the analysis of potential needs of the customers according to their asset status, business situation, and industry characteristics, fully consider the expected development needs of customers, improve the investigation contents of credit matters, report multiple matters at the same time, and conduct a combined investigation on credit business matters, which can effectively reduce the repeated investigation and report of multiple credit businesses for a single customer, and improve the efficiency of business operation. At the same time, agricultural land financial institutions should carry out policy financial business with the support of the state, and provide long-term and low-interest loans to farmers through the innovation of financial products and financial instruments, to realize the strategic goal of agricultural modernization. Especially for Heilongjiang Province, focusing on the national and provincial “three rural” policy guidance, combined with the characteristics of agricultural development in Heilongjiang Province, we should innovate the personal credit products of three rural areas, and gradually establish the personal credit product system of three rural areas suitable for family farms. We have innovated loan varieties suitable for Heilongjiang family farm and other new agricultural business entities designed a set of risk controllable and easy to operate process specifications for serving new agricultural business entities, and made full use of order agriculture, “company + farmers,” “credit company + farmers,” “leading enterprise + base,” and other forms to innovate family farm loan business mode.

Family farm operators promote the quality of farmers by building training centers for the farmers or combining agricultural colleges and family farms. Enhance the subjective initiative of operators to learn financial knowledge, so that operators can actively understand the financial information about benefiting farmers launched by banks and cooperatives in various regions, and pay attention to credit policies conducive to their financing from various aspects and channels. At the same time, it is necessary to cultivate the sense of integrity of the operators. After obtaining the loan, the operators must strictly use the special funds for a special purpose, to achieve a “win-win” result for both sides.

## Data Availability Statement

The original contributions presented in the study are included in the article/supplementary material, further inquiries can be directed to the corresponding author/s.

## Ethics Statement

Ethical review and approval was not required for the study on human participants in accordance with the local legislation and institutional requirements. Written informed consent from the participants was not required to participate in this study in accordance with the national legislation and the institutional requirements.

## Author Contributions

HW: conceptualization and formal analysis. SZ: writing the original draft. JG: conceptualization. YF: validation. All authors contributed to the article and approved the submitted version.

## Conflict of Interest

The authors declare that the research was conducted in the absence of any commercial or financial relationships that could be construed as a potential conflict of interest.

## Publisher’s Note

All claims expressed in this article are solely those of the authors and do not necessarily represent those of their affiliated organizations, or those of the publisher, the editors and the reviewers. Any product that may be evaluated in this article, or claim that may be made by its manufacturer, is not guaranteed or endorsed by the publisher.
